# Residual Stress Enhancement by Laser Shock Treatment in Chromium-Alloyed Steam Turbine Blades

**DOI:** 10.3390/ma15165682

**Published:** 2022-08-18

**Authors:** Festus Fameso, Dawood Desai, Schalk Kok, Dylan Armfield, Mark Newby

**Affiliations:** 1 Department of Mechanical and Mechatronics, Tshwane University of Technology, Pretoria 0183, South Africa; 2 Department of Mechanical and Aeronautical Engineering, University of Pretoria, Pretoria 0002, South Africa; 3 Eskom Holdings SOC Ltd., Johannesburg 2001, South Africa

**Keywords:** finite element, laser shock, residual stress, surface treatment, turbine blade

## Abstract

In-service turbine blade failures remain a source of concern and research interest for engineers and industry professionals with attendant safety and economic implications. Very high-pressure shock impacts from laser shots represent an evolving technique currently gaining traction for surface improvement and failure mitigation in engineering components. However, the physical characteristics and effects of parameter variations on a wide range of materials are still not fully understood and adequately researched, especially from a computational point of view. Using the commercial finite element code ABAQUS©, this paper explores the application of laser shock peening (LSP) in the enhancement of residual stresses in Chromium-based steel alloyed turbine blade material. Results of the numerically developed and experimentally validated LSP model show that peak compressive residual stresses (CRS) of up to 700 MPa can be induced on the surface and sub-surface layers, while the informed varying of input parameters can be used to achieve an increase in the magnitude of CRS imparted in the peened material. Analysis of the hierarchy of influence of the five input parameters under investigation on residual stress enhancement reveals the laser shock intensity as the most influential, followed in descending order of influence by the exposure time, shot size, degree of overlaps, and the angle of shot impact.

## 1. Introduction

From the design to the manufacture and maintenance of engineering components, the interest in how residual stresses influence the properties, utility, and life cycle of equipment parts, such as turbine blades, continues to grow substantially. Knowledge in this area can be applied in the structural assessment of the combined effects of in-service and internal stress states on the safety and failure-resistance of manufactured components, bearing in mind the structure-process-property relationships on eventual performance post-commissioning. As a result, concerted efforts in terms of research and engineering are being committed to the innovation of frameworks that incorporate or induce residual stresses into components, be it at the design stage or at the maintenance or re-manufacture stages. The severity of stress distribution around a crack initiation site is represented by a stress intensity factor ∆K below which no crack propagation can occur, hence in other to mitigate fatigue and delay or outrightly prevent failure, the values of ∆K have to remain as low as possible. This is possible by reducing the effects of tensile stresses which feed crack initiation and propagation with equal or more, but opposite compressive stresses. According to [1], when a tensile stress field is superimposed onto applied load cycles, the effective stress intensity factor becomes higher. On the other hand, according to the analysis of [2], compressive residual stresses superimposed on the applied stress field effectively lower the stress intensity factor, hence preventing tensile stress-induced failure.

**Figure 1:**
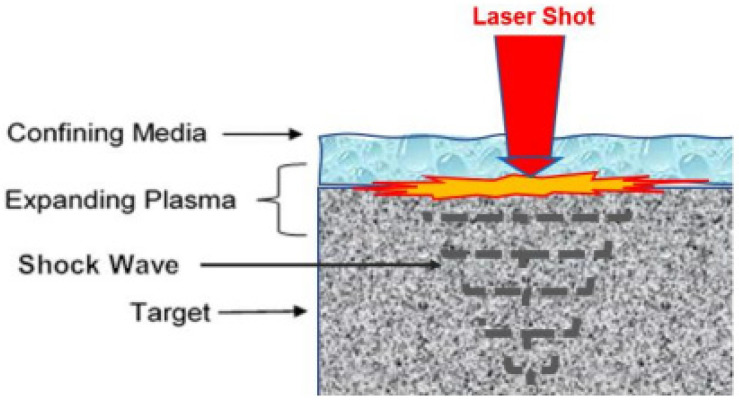
The confined laser shock peening process.

Laser shock peening (LSP) is used in modifying some mechanical properties of alloys just like treatment methodologies such as steel aging. However, unlike steel aging which is carried out at elevated temperatures of up to 250 °C and extended durations of 500–1500 h [3], LSP imparts residual stresses in materials by dynamic shock impact of impinging high intensity, gigawatts per square centimeter range, laser shots, on the surface of materials at room temperature or below, occurring in nanoscale durations. As shown in Figure 1 [4], in their study of laser-induced waves with metals, explained the concept of LSP as laser shots create rapidly expanding plasma from the ultra-fast (less than 1 microseconds) vaporization of the micron-thin layer of the surface of the target. In the process, pulses of high-pressure shock waves are produced, which when greater than the yield strength of the material under shock, cause plastic deformation and induction of stresses in the direction of propagation of the shock waves. Subsequently, compressive residual stresses remain in the part, improving damage tolerance, fatigue strength, and ultimately, the useful life of the material by counteracting the effect of applied tensile loads and stresses.

The potential of LSP as a surface engineering process of choice has over time been a subject of several investigations, due to its offering prospects of possible direct integration into manufacturing, high degree of automation, increasing quality assurance, treatment of localized fatigue critical areas without masking, and making design changes that would not require additive or subtractive production. Most of the earlier investigations reported by [5,6,7], mostly by experiments, have shown high amplitude stress waves could be generated in materials by impinging the laser beam on an unconfined surface of the body and vaporizing a small amount of surface material. Later works by [8,9,10] have also involved direct measurements of pressure improvement of the induced pressure, and stress profiles have also shown promise. In the last decade or two, the development of more robust and advanced computing has expanded the perspectives of LSP studies, with finite element modeling and simulation of LSP-induced residual stresses time and again reported in literature, mainly under axisymmetric conditions. Refs. [11,12,13,14,15,16,17,18,19,20,21,22,23] have at different times, using different methodologies and material models, applied FE simulation to determine the constitutive behavior and response of a range of materials to laser-impacted conditions.

However, limited work has been done in terms of parametric investigations of the response and effects on chromium-based steam turbine blade materials, variation of input parameters, parameter testing and/or sequence of input factors, and the hierarchy of influence of these factors on residual stress enhancement applications of LSP in engineering materials, particularly as it applies to failure prevention and useful-life improvement of turbine blades. Furthermore, most of the erstwhile studies have not incorporated fully physics-based constitutive behaviors in modeling the high strain rate evolution characterizing LSP as well as the hydrodynamic behavior and contribution of confining fluid medium under shock conditions. In a distinctly different approach compared with previous studies, this study uses a FEM based approach, coupled with a fully physics-based mechanical threshold stress (MTS) model; described by [24], which accounts for the stage-wise hardening and strain rate jumps highlighted by [25] characterizing high strain-rate deformation associated with LSP; with an equation of state hydrostatic stress tensor description of shock wave and experimentally described laser loading profile, to predict and enumerate the effects of variation of five important LSP input parameters. These parameters include shock intensity, degree of overlaps, shot size, shot angle, and exposure time, varied at different magnitude ranges. An investigation was carried out on their influence on the evolution of the residual stresses induced in a 12% Chromium based martensitic steel used in the manufacture of steam turbine blades. Further considered was the analysis of the hierarchy of influence on these parameters on the generated residual stresses, in a bid to establish laser peening rule frameworks for the set up of the perfect peening state that will bring about the best execution of LSP for condition-based maintenance and structured health monitoring of power generation equipment.

## 2. Materials and Methods

This research employs numerical modeling and simulation, using the commercial FE software ABAQUS^®^ to establish the residual stress enhancement frameworks for the industrial application of LSP as practicable and effective manufacturing, re-manufacturing, and maintenance process. This makes it possible to investigate the effect of changes in the operating parameters without producing a physical prototype. During this process, it becomes possible to explore the merits of these parameters, and to make inferences from the results obtained, thereby reducing the cost and time to be spent. The two- and three-dimensional visualization of the results will also allow for easier verification, communication, and understanding of the problem and the results, both in real-time, and when the research is eventually concluded, thus strengthening designs where vulnerable without necessarily adding material or weight.

### 2.1. Subsection

**Figure 2:**
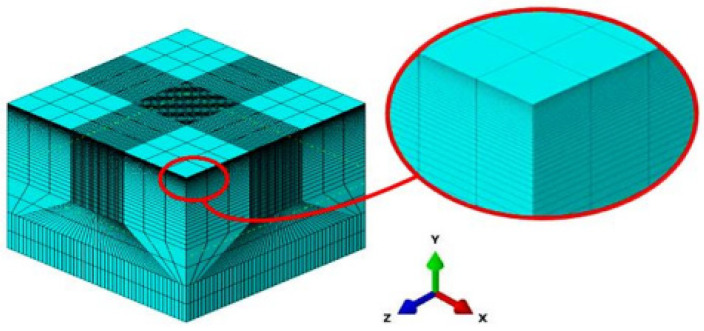
The discretized finite elements mesh of the geometric model.

A three-dimensional geometric model of a cut-out section of a turbine blade was defined employing the geometry part building blocks native to the complete Abaqus environment of the commercial software ABAQUS^®^. As shown in Figure 2, the geometry of dimension 20 mm by 20 mm by 10 mm was created with the attributes of a solid, homogenously deformable component.

It was discretized into finite elements using the mesh module, using structured meshing techniques as prescribed by [26], into first-order linear brick hexahedral continuum elements with reduced integration (C3D8RH) and hourglass control, with the use of meshing bias function in the axial direction to create a denser meshing at the layers close to, and beneath the top surface. Top face partitions array subsections of 0.01 mm were integrated on the peened area to ease the imposition of the stepped pressure function and load definition as well as generation of appropriate meshing for the multi-stepped analysis for which the geometry was being designed. Following mesh convergence analyses, a refined mesh seed size of 0.08 mm was assigned, local to the peened area, being the area of interest. Acoustically silent infinite boundary elements were applied to the outer boundaries of the geometry as non-reflecting boundaries that limit the effects of reflection, interaction, interference, and amplification of propagating shock waves. Zero displacement mechanical boundary conditions in all coordinates were thereafter applied to the boundaries of the geometry to impose non-depthwise displacement effects on the boundaries of the target during analysis.

### 2.2. Loading Conditions

**Figure 3:**
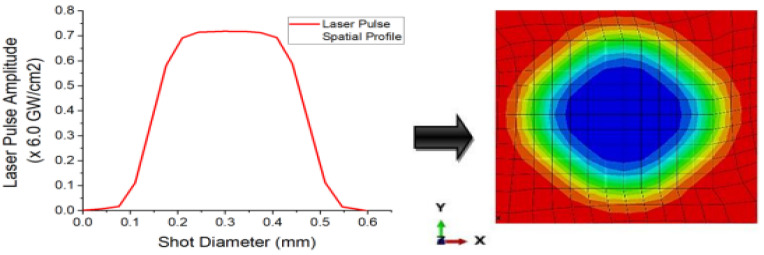
Laser shot spatial intensity distribution profiles.

The loads attributable to the laser shots, which manifest as plasma-induced shock waves were defined by pulses of high magnitude pressure applied to partitioned sections of the geometric model. In line with contact analyses, the shock loads were defined as discontinuous constraints impacts that allow transmission and propagation of the pressure forces from one unit of the discretized geometry to the other, until the analyses of the damping of the forces have been fully computed within the analysis time frame imposed. The pressure loading description is defined with respect to spatial (space), as shown in Figure 3, and temporal (time) profiles defining the size and intensity of the shot and a given period, across its intended area of coverage.

Table 1 provides further loading parameters applied in the development of the simulation model for the LSP analysis.

The time-dependent loading profile was adapted from [27]’s pressure determination with the loading amplitude being a function of the peak pressure, to simulate the laser shock impacts and hence the mechanical shock wave propagation and the residual stress generation in the workpiece. Following [28,29]’s semi-empirical formula of shock wave pressure together with the assumption that the incident laser power density, and the resultant shock wave pressure distribute uniformly within the laser spot. For a constant absorbed laser intensity $I_{o}$ and a laser pulse duration, τ, the peak of shock wave pressure, P_max_, is written as:

(1) $$P_{\max}=0.01\sqrt{\frac{\alpha }{2\alpha +3}}\cdot \sqrt{\frac{2{\tilde{z}}_{1}{\tilde{z}}_{2}}{{\tilde{z}}_{1}{\tilde{z}}_{2}}}\sqrt{I_{o}}$$

Which eventually reduces to:

(2) $$P_{\max}\;(\mathrm{GPa})=1.6\sqrt{I_{o}}\;({\mathrm{GW}/\mathrm{cm}}^{2})$$

where $\alpha ,\;{\tilde{Z}}_{1},$ and ${\tilde{Z}}_{2}$ represent the energy fraction of the laser shock, the shock impedance of the target material, and the shock impedance of the confining media respectively.

### 2.3. Materials and Constitutive Modelling

**Figure 4:**
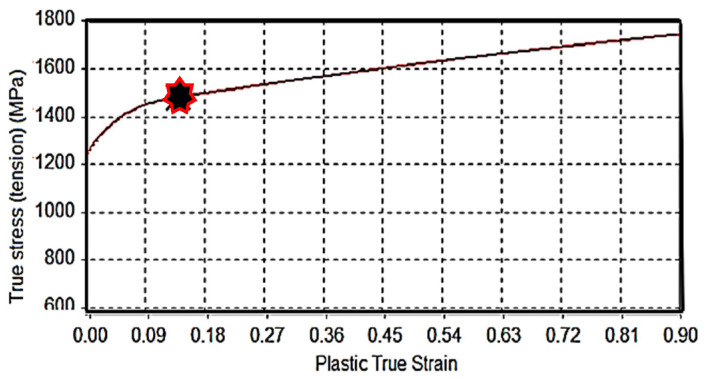
True stress-strain curve (in tension) of X12Cr alloyed steel with (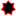) indicating the onset of necking [30].

Metal behavior at high strain rates exhibits a non-linear response of applied loading, referred to as plasticity. This nonlinear behavior is defined in conjunction with its elastic and hydrostatic behavior to model the dynamic response of the chromium-based steel alloy material of the turbine blade, to shock impact and plasma pressure propagation as experienced during laser shock peening. At the material’s mechanical elastic limit, where the mechanical deformation of the material becomes totally unrecoverable, yield, hardening, and accumulation of elastic and plastic strains place restrictions on its shear behavior. Noted for its good corrosion and fatigue resistance properties, X12Cr stainless steel in its martensitic state is an ideal material for use in the manufacture of turbine blades. Table 2 and Table 3 and Figure 4 provide some material properties of X12Cr steel.

**Figure 5:**
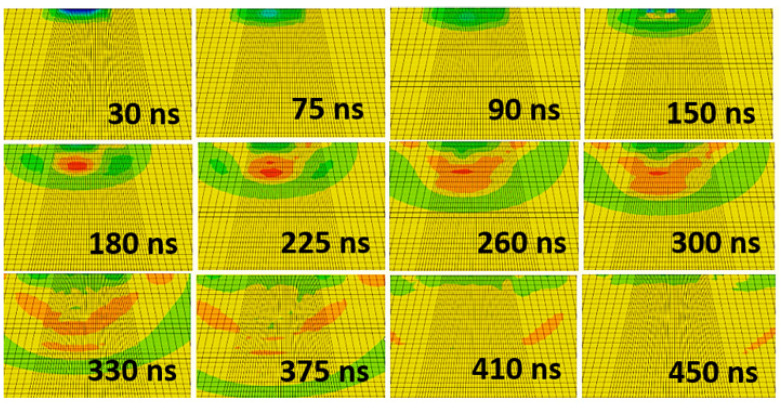
Propagation and attenuation of induced shock waves with respect to time.

In accurately modeling the physics of the laser shock impact under a water-confined regimen, intentional consideration has to be given to accounting for both the elastoplastic responses and the hydrodynamic contribution to the build-up and attenuation of the shock, with or without work-hardening of the material. This involves the coupling of the constitutive laws which govern the development of the plasma pressure from the instantaneous ablation and vaporization of the target’s surface at laser-shock impact, accumulation of the pressure to magnitudes beyond the plastic regime of the material to induce material deformation, the full physics of the deviatoric material plasticity at ultra-high strain rate and the hydrodynamic equation of state that accounts for the volumetric stress component of the resultant stress tensor. Thus, together with the mechanical effects induced, the fluid-like behavior of shock wave as it generates and propagates, transferring plastic energy into the material, is reproduced in the simulation model, as shown in the attenuation response in Figure 5.

Using the elastic-perfectly plastic (EPP) model, which provides a formulation for estimating the amount of stress that would cause yield in the material as a result of shock waves propagation, the magnitude of the stress that will graduate the deformation from a typical elastic deformation to a perfectly plastic deformation is obtained from [15] as:

(3) $$\sigma_{y}=HEL\frac{1-2v}{1-v}$$

where $\sigma_{y}$ is the dynamic yield strength, v is the Poisson’s ratio and HEL represents the Hugoniot elastic limit of the material. The mechanical threshold stress (MTS) constitutive model, a deviatoric stress measure, developed by [32] was applied to replicate the inelastic response in the material. The MTS model is a constitutive, fully physics-based model, based on material plasticity with full path dependency on state variable evolution. It is scaled such as to account for the strain rate and temperature dependencies of the material during plastic deformation, to obtain the material flow stress. The threshold stress is the flow stress of the material, for a given internal structure, at 0 K. This is the stress required to overcome obstacles in the absence of any contribution from thermal activation energy characterizing the strength and density of obstacles. It is decomposed to reflect a thermal component and an athermal component and is given by [31] as:

(4) $$\sigma_{y}(\varepsilon_{p},\dot{\varepsilon_{p}},T)=\sigma_{a}+\sum_{k}\sigma_{t}^{k}$$

where $\sigma_{y}$ represents the material flow (or yield) stress, $\sigma_{a}$ represents the athermal stress component, and $\underset{k}{\sum }\sigma_{t}^{k}$ represents the thermal component of the model. The thermal component is expanded further as:

(5) $$\sum_{k}\sigma_{t}^{k}=(S_{i}\sigma_{i}+S_{e}\sigma_{e})\frac{\mu (P,T)}{\mu_{0}}$$

where $\sigma_{i}$ represents the intrinsic component of flow strain due to barriers to thermally activated dislocation motion, $\sigma_{e}$ is the strain hardening component of the flow stress. $S_{i}\;\mathrm{and}\;S_{e}$ are the strain rate and temperature-dependent scaling factors and μ and $\mu_{0}$ are the shear modulus, and the absolute zero shear modulus, respectively. This hydrodynamic behavior was formulated using the Mie–Gruneisen shock equation of state (MGEOS) in which the ratio of pressure to specific energy is independent of temperature. It captures the pressure contributions due to hydrostatic compaction of the water-confined medium, in the shock-compressed solid target. The MGSEOS is mathematically expressed as:

(6) δP=f(ρ(δe))=Γρ(δe)

In relation to a reference state:

(7) $$\left(P-P_{ref})=\Gamma \rho (e-e_{ref}\right)$$

where the pressure P is generally referred to as the hydrostatic stress in a stress tensor, ρ is the material’s density and e is the specific internal energy of the system. $P_{ref}$ and $e_{ref}$ both represent reference pressure and internal energy states. Equation (7) evaluates as:

(8) $$(P-P_{ref})=P_{h}=\frac{\rho_{o}C_{o}^{2}\eta }{{\left(1-\frac{\eta (1+\Gamma_{o})}{2}\right)}^{2}}$$

with Equation (8) being the Hugoniot pressure $P_{h}$, and

(9) $$(e-e_{ref})=e_{h}=\frac{1}{2}P_{h}(v_{o}-v_{1})=\frac{P_{h}\eta }{2\rho_{o}}$$

and Equation (9) being the Hugoniot energy $e_{h},$ i.e., the difference between the specific internal energy ahead of, and behind the shockwave. Thus, the hydrostatic pressure relating to the Mie-Gruneisen shock equation of state (MGEOS) becomes fully defined as:

(10) $$P=\frac{\rho_{o}C_{o}^{2}(\eta -1)[\eta -\frac{\Gamma_{o}}{2}(\eta -1)]}{{\left[\eta -S(\eta -1)\right]}^{2}}+\Gamma_{o}E$$

where S, $C_{o}^{2}$, η, $\Gamma_{o}$, and E=ρe represent the Hugoniot slope coefficient, speed of sound in the material, volumetric compressive strain, Gruneisen constant, and total internal energy respectively. The parameters for Equation (10), obtained from [30] are presented in Table 4 for X12Cr alloyed steel and applied using the ‘EOS’ tab in the linear hugoniot form Us-Up.

### 2.4. Analysis Steps

**Figure 6:**
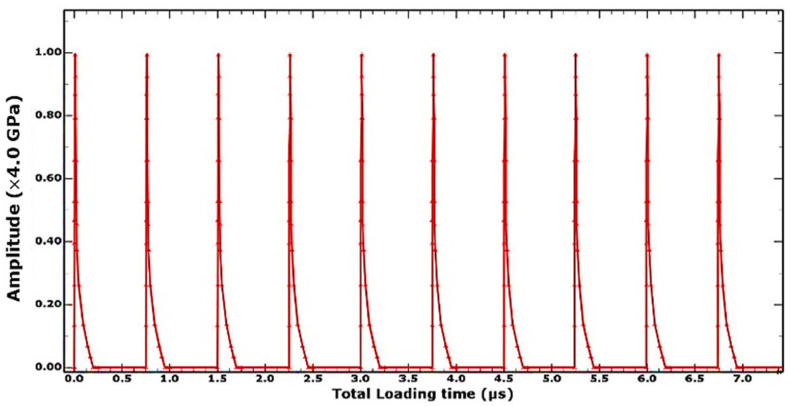
Single-explicit multiple-stepped analysis profile.

Being a nanoscale operation characterized by the impact and response of the laser-shot load on a metallic target, occurring within the space of milliseconds as a quick, sharp, sudden impact followed by internal stress distribution, numerical LSP computation is predicted from a complex loading regime, with variations in the temporal and spatial functions of the load applications, a nonlinear elastic-plastic explicit time integration suits its short-duration transient analysis and computation. The explicit analysis method developed by [24] was applied to simulating the stepwise loading and unloading of the laser shots on the material as illustrated in Figure 6. General dynamic explicit steps, with the response per step specified for loading type, amplitude, propagation, and timed damping, were imposed, with the starting point for each step being the state of the model from the end of the previous step.

**Figure 7:**
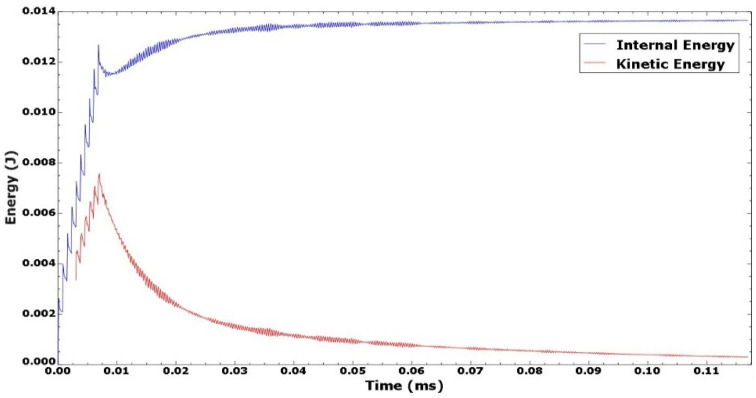
System dynamic response and energy field profile.

The first explicit dynamic step computes the shock loading of the discretized geometry within the spatial and temporal domain typified by the loading condition described in Section 2.2. The impact, induction, and propagation of elastic and plastic shock waves evaluated within a step-time of 250–500 ns determined by the observed time it takes for all the plastic waves to attenuate, leaving only elastic shock waves which aren’t potent enough to cause any plastic deformations in the material. The sequence of loading and unloading steps combine to form a simulation and analysis of multiple laser shots upon the specified peen area. An extended no-load final dynamic explicit step three orders of magnitude higher (at 0.1 milliseconds and above) than the previous steps accommodates the damping phase of the overall shock wave attenuation, where the energy within the system dissipates to less than a percent of its shock state, resulting in a stable stress and energy field within the system as shown in Figure 7. The induced stresses at this quasi-static state are what obtains as the residual stress, usually compressive in nature unless under extreme shock conditions. As reported in the study by [20], the extended final damping step de-excites the system to an equilibrium state and addresses the constraints of computational cost and time, providing a quicker solution clock-time. The computed stress and strain field distribution within the material can thence be obtained at the end of this analysis step.

## 3. Results

**Figure 8:**
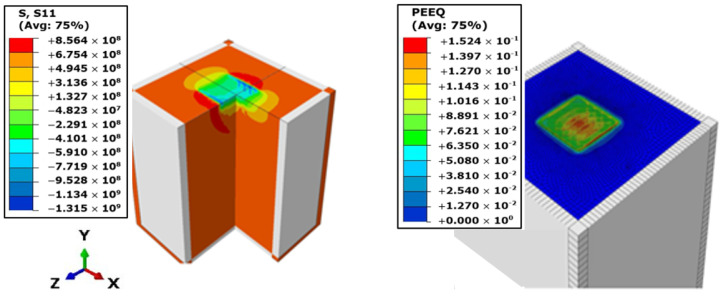
3D simulation visualization of the stress (S11) and equivalent plastic strain (PEEQ) distribution results of the peened geometry.

Having developed the finite element model template to be employed in undertaking the parametric investigation of effects of LSP input factors on residual stress generation, the simulation was run on Intel Core i5 7th generation computer with quad-core processors and 16 GB of RAM, running on 64-bit Windows 10 operating system. The simulation time for every simulation run averaged 102 min of clock-time, with an average of 25 GB of disk storage incurred in output files generated per analysis. Figure 8 presents the simulated results of the depth and surface distribution of surface residual stress and equivalent plastic strain for the FE simulation. The simulation was carried out with 6 GW/cm^2^ shot intensity, shot sizes of 0.6 mm normal to the surface of the target, and overlapping at 83% degree of overlaps. The full-width half maximum (FWHM) peak of the shot exposure time was at 12.5 ns for a step-time of 250 ns. The results were taken at the end of the extended final relaxation step when dynamic equilibrium had been achieved and principal stress fields were safe to be considered as being residual. The colored regions represent laser shot affected regions (LSAR) of finite nodes and boundaries, within whose domains the stress, strain, and displacement profiles are computed. The grey regions constitute the boundaries of the geometric model, which are made up of infinite boundaries that absorb shock waves impinging on them from the propagation laser shot’s stress waves and not reflecting them into the system to cause interference, which may alter the accuracy of the results.

Peak compressive stresses of up to 1.315 GPa were obtained at the surface which paled with depth into the target material to plastically affected depths of about 0.6 mm. Peak equivalent plastic strains of up 0.152 were recorded at the surface mostly at regions of successive overlaps of the laser shots. The result was verified with analytical calculations, using Ballard’s empirical formulation presented by [33] for the maximum induced surface stresses in compression by a fast laser shock impact on an elastic-perfectly plastic material. Using the equation:

(11) $$\sigma_{surf}=-\frac{P}{2(1+\frac{\lambda }{2\mu })}[1-\frac{4\sqrt{2}}{\pi r}(1+\nu )\frac{C_{el}C_{pl}}{C_{el}-C_{pl}}\tau ]$$

where the equivalent peak shock pressure, P = 4 GPa, the radius of the laser shot, r = 0.3 mm, material’s poisons ratio, ν = 0.3, and the FWHM peak exposure time *τ* = 12.5 ns. λ and μ are material constants referred to a Lame’s constants, evaluated as:

(12) $$\lambda =\frac{\nu E}{\left(1+\nu )(1-2\nu \right)}=118\;\mathrm{GPa}\;\;\;\mathrm{and}\;\;\;\mu =\frac{E}{2(1+\nu )}=79\;\mathrm{GPa}\;$$

where E = 204.2 GPa is the young’s modulus of elasticity of the material.

The elastic wave speed $C_{el}$ is obtained from $C_{el}=\sqrt{\frac{3K(1-\nu )}{\rho (1+\nu )}}$ to be equal to 6000 m/s. Similarly, the plastic wave speed $C_{pl}$ is obtained from $C_{pl}=\sqrt{\frac{K}{\rho }}$ to be equal to 4726.3 m/s. K = 172 GPa, is the bulk modulus of the material in compression, and ρ = 7700 kg/m^3^ is the material density. Thus, the maximum induced compressive surface stress is evaluated as:

(13) $$\sigma_{surf}=1.342\;\mathrm{GPa}$$

**Figure 9:**
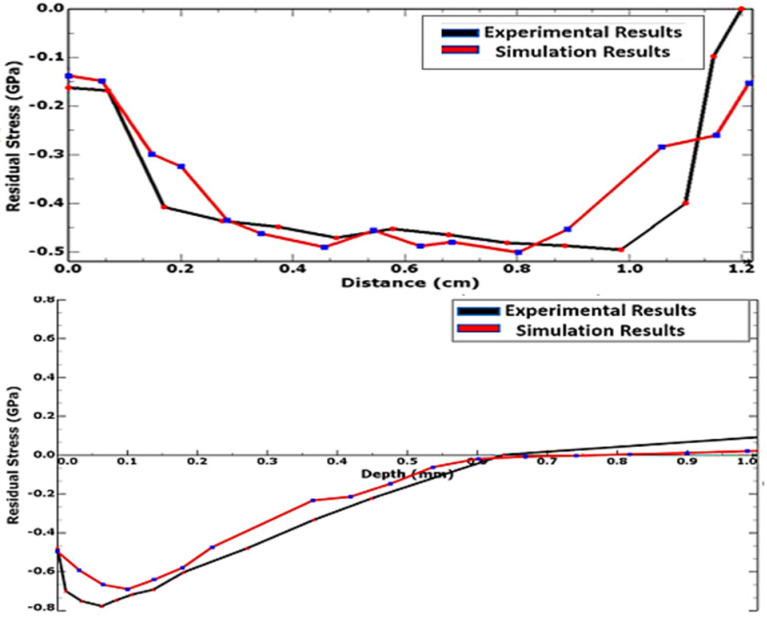
Numerical vs. experimental results benchmarking.

Benchmarking the simulated and analytically evaluate max surface stress, the variance is obtained to be at 2.04%, indicating a close correlation of the LSP FE model developed. In a similar vein, the simulation model was experimentally validated, with the in-plane and in-depth compressive residual stress (CRS) distribution profiles benchmarked with experimental results from the LSP experiment carried out under controlled conditions at the National Laser Centre (NLC) of the Centre for Scientific and Industrial Research (CSIR) and published in the work of [31]. The depth and distribution of residual stresses, measured as the radial ($\sigma_{xx}$ or $S_{11}$) variation of residual stress with depth, for both the benchmark experiment and the FE simulation are presented in Figure 9.

The simulated residual stresses are compressive, reaching a maximum of about 715 MPa at 0.07 mm beneath the surface, from 480 MPa at the surface, and penetrating as deep as 0.67 mm into the material. These values reveal a close correlation to those obtained from the experimental benchmark. Table 5 provides a summary of the comparison of the simulated and experimental results.

This lends credence to the LSP model, including its geometric, constitutive, and analysis parameters, showing very good agreement and within the recommended range of <10% variance for the reliability of model results. Having validated the LSP FE model with experimental results and an appreciable level of correlation and validity established, the model was implemented in the parametric investigation of the effects of linearly increasing the magnitudes of the various input parameters on the outputs.

## 4. Discussion

### 4.1. Effects of Laser Intensity

**Figure 10:**
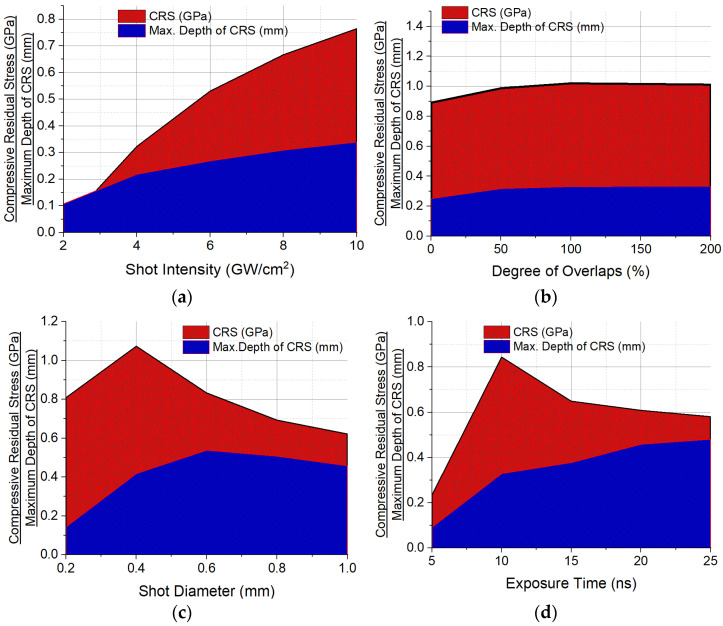
Parametric investigation of the effects of linearly increasing/decreasing magnitudes of the input parameters on residual stress enhancement. (**a**) Laser shot intensity. (**b**) Degree of shot overlap. (**c**) Shot diameter. (**d**) Exposure time.

Simulations were carried out for laser shock intensities within the range of 2 GW/cm^2^ to 10 GW/cm^2^ prevalent in practical LSP applications. Irradiated at constant input parameters, bar the laser shot intensity, the residual stress field is found to respond to an increase in shot intensity with an increase in the magnitude of compressive residual stress induced in the target. As illustrated in Figure 10a, higher magnitudes of compressive residual stresses (CRS) and penetration depths ($d_{p}$) were recorded for every increase in laser shot intensity, asymptotically saturating at up to 800 MPa of CRS. The most significant increases were found between shot intensities of 2 GW/cm^2^ and 6 GW/cm^2^, which may be a good indication of the range of economic viability of the application of increasing shot intensity to residual stress enhancement, be it in manufacturing, maintenance, or remanufacturing of the turbine blade material under investigation.

These trends are in tandem with observations from experimental studies of similar nature. Ref. [34] reported that CRS could be increased and also driven deeper below the surface of the metal target by increasing the shot intensity until a certain power intensity threshold is exceeded, when saturation begins to occur. Ref. [35] reported similar observations in aluminum alloys and stainless steel.

### 4.2. Effects of Superimposing Laser Shots

It is commonplace in practice to either arbitrarily or in defined patterns, peen parts repetitively at specific regions, repeating the sequence and intervals as the target or laser beam is translated. The investigation of superimposed shot effects has shown to alter the residual stress distribution profile in the peened area. Observed at regions of the intersection of the overlapping shots, with 0% indicating no lapping and 100% indicating a fully superimposed shot pattern, the CRS profile experienced an increase in magnitude, as shown in Figure 10b, until stress saturation begins to occur at multiple impositions of shots on the same spot beyond twice. The most significant increase was observed between 0% to 50% degree of overlap, indicating the desirability of half to full shot laps in delivering increased magnitudes of CRS to deeper penetration depths when other input parameters are stable or unvaried. These observations are not far-fetched from those of [36,37,38] who all reported a peak residual stress increase which later began to decrease as the degree of overlaps continued to increase, and surface deformation increases with the number of overlaps but tends to saturate as the number of repetition increases.

### 4.3. Effects of Shot Diameter

The inadvertent change in the beam density at constant energy when the focus size of the lens through which a beam passes is altered has been observed to impact the residual stress distribution of a laser-shot treated material. From Figure 10c, results reveal increased CRS with the reduction in shot diameter (until the focus size becomes extremely relatively small and less luminosity hits the target) with a corresponding increase and then decrease in plastically affected depth. Largely due to the inverse proportionality of spot size and power intensity, as explained by [39], smaller spots generate higher beam densities which will, in turn, induce higher CRS until the threshold of the focal lens characteristics is reached when the size of focus begins to diminish the strength of the beam impacting the target. Increasing focus sizes steadily changes the profile, direction, and energy of attenuation of shock waves, producing more planner propagation of shock waves, which drive plastic energy deeper into the material until a threshold is reached where the quantum of the shock waves spreading out in both axial and radial planes dissociate much earlier in its propagation, thereby losing energy required to achieve deeper depths of penetration. This accounts for the increase and subsequent decrease in depth of CRS penetration. Similar results have been obtained by [37,40,41], who all reported compressive stresses to decrease as the shot diameter increases and surface deformation increase and then decrease with an increase in shot diameter.

### 4.4. Effects of Peak Pressure Exposure Duration

The duration taken for peak pressure to be attained from the beginning of exposure to laser intensity, being a function of amplitude versus step time of the action of pressure in a timed domain has been found to contribute effects to the eventual distribution of residual stress post-peening operation. As shown in Figure 10d, the temporal distribution of the shock wave pressure generated to increase the depth of penetration of CRS, with this effect being significant with long laser pulse peaks of between 10 ns to 20 ns. Conversely, the magnitude of the CRS decreases with increasing pulse peak duration. This is only after an initial jump at very short pulse durations below 10 ns. The interaction between the corresponding development and growth of the pressure quantum and the time available to grow it can be responsible for this. Inadequate time for the development and growth of the pressure plasma after the laser-shot induced vaporization of the material’s surface localizes plastic deformation and does not generate enough plastic waves to propagate. At relatively longer durations, CRS decreases with increasing FWHM peak durations largely due to the extended periods of irradiation, which creates more than enough time for energy deposition and diffusion before the shockwaves that induce the residual stresses begin to propagate. Recall LSP is a nanoscale operation that relies on the speed of rapid shock and expansion of high-pressure plasma to drive home plastic deformation and compressive residual stress in the material. The residual stress field depth of penetration begins to saturate though, at much longer peak durations above 25 ns. These outcomes align with the outcomes of studies conducted by [28,42], who demonstrated in their studies that shorter peak pressure pulse durations increase SRS but result in shallow depths of CRS penetration while longer pulses result in deeper penetration and that the plastically affected depth is controlled by the pulse duration, with a long pulse inducing a greater depth of compressive residual stress.

### 4.5. Effects of Angle of Shot Impact

**Figure 11:**
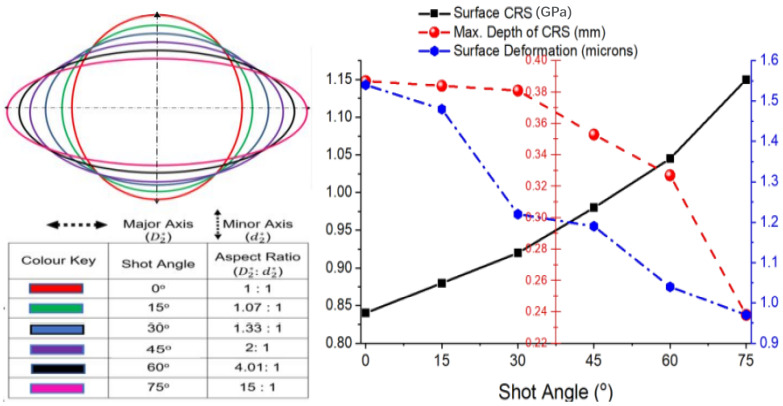
Variable shot angle effects on residual stress enhancement.

While prevalent LSP applications present the peened workpiece at a perfectly normal angle of treatment to the direction of the laser shots, inclined peening angles which require laser peening at angles other than normal to the peened surface are unavoidable, especially when treating slots, keyways, grooves, and other complex and irregular geometries with twists and pitches (such as found in turbine blades). The inclined angle of incidence of the laser shots as it changes from normal to the surface of the impact causes the laser spot to spread over a larger diameter at aspect ratios which varies the geometry of the shot from the perfect circular geometry of a circular shot impacting at an angle normal to the surface of impact. Modifications in spot geometry in terms of aspect ratio and refraction of the laser beam at the material interface alter the total surface energy per unit area at constant beam density. From Figure 11, CRS is observed to increase with the increase in shot angle, as the total energy increases with the increasing shot angle. The pressure input per unit area continues to experience and increase as the axis of the emanating elliptical beam geometry constricts within the spatial domain in response to changing aspect ratios. With the corresponding alteration of the shock wave attenuation function, dissipating over a larger spatial domain in both axial and radial planes, low-energy shockwaves transit between interfaces with varying densities at inclined angles resulting in decreased depth of penetration as the laser irradiation incidence angle increases, becoming more prominent as refraction effects become more significant at angles greater than 30°.

Thus far, the variational effects of increasing laser shot angles on LSP outcomes have been sparsely investigated and are quite limited in literature. However, ref. [43] reported from experiments that near the peened surface, varying the incidence from the normal angle introduces anisotropy with the stress in the radial direction becoming more compressive as the angle of incidence increases.

### 4.6. Analysis of Hierarchy of Influence of LSP Input Factors on Residual Stress Generation

**Figure 12:**
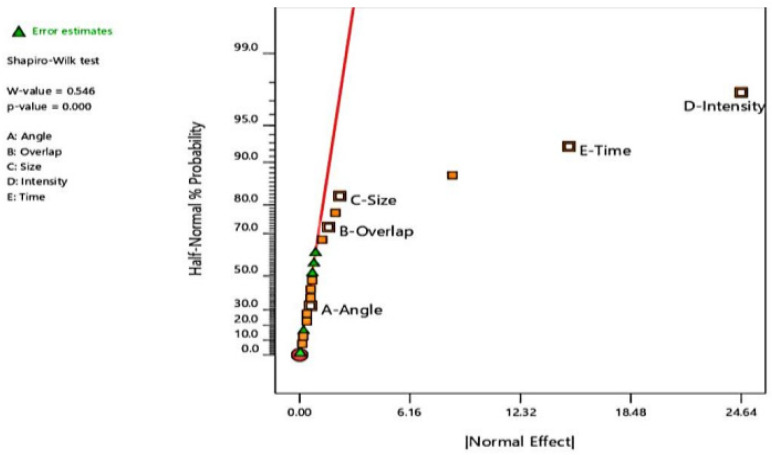
Half-normal percentage probability analysis plot for residual stress response.

The effects and influence of the 5 input factors under consideration in this study were evaluated by statistically analyzing the simulated results of thirty runs of random combinations of input parameters. The normality of the residual stress results processed from the simulation runs was computed using the Shapiro–Wilk normality test based on the Shapiro–Wilk hypothesis, which was applied in quantifying and weighing significant departures of the outcomes, from the distribution. The outcome of the analyses is presented in Figure 12, in form of half-normal probability plots for each of the design responses. The plot displays the absolute value of the effect estimates ordered against their cumulative normal probabilities, from largest to smallest. The laser shock intensity (D) and the FWHM peak exposure time (E) are furthest away from the reference line, with the highest values of absolute effects. This indicates the strength of their significance to the process and shows how much they influence the induction of residual stresses, with the laser shock intensity showing the most influence.

The shot size or diameter (C), degree of overlaps (B), and shot angle (A) bring up the rear in that order, with the shot angle the least significant in its influence on residual stress induction and enhancement compared to the remaining input factors. This information is tabulated in Table 6.

## 5. Conclusions

This study has presented the development of a numerical, experimentally validated laser shock peening simulation model, based on a finite element computational procedure, which can be successfully applied to the prediction of the residual stress regime of a chromium-based martensitic steel alloy material suitable for use in the manufacturing of power plant components like steam turbine blades. The coupling of the physical aspects of the constitutive behavior of the material under a confined laser shock loading regime took into account the definition of the process of peak plasma pressure build-up, the plastic regime, strain-rate jump at ultra-high strain rate associated with laser shock treatment, as well as the hydrodynamic contribution to the build-up and attenuation of the shock, with or without work-hardening of the material. The simulations were verified analytically and validated with benchmarks from an experiment carried out with equivalent parameters, showing very good correlation and lending credence to the validity and suitability of the numerical model for the prediction of the enhancement of residual stress in the steam turbine blade material and the parametric study of the effects of variation of input parameters on laser-peen induced residual stress induction in the material.

Consequent to the aforementioned, the results returned during this study have revealed that in subjecting materials/components to laser shock treatment, the residual stress distribution can be controlled to desired dimensions by controlling the different input factors. Achieving greater magnitudes of CRS can be controlled by increasing, within ranges prescribed in this study, input parameters of factors, such as the intensity of the laser beam, degree of overlap of the shots, as well as the angle of attack to the normal. The opposite will be the approach for factors, such as the size of the shot and the duration of exposure time the target is irradiated by the laser beam for the peak pressure, to be attained. In the same vein, a very short pulse duration or too small a shot size may have unsatisfactory effects on LSP outcomes. In order of influence of residual stress enhancement in peened materials, the shot intensity has been determined to be the most influential LSP input factor, showing the highest normality and sensitivity of the factors considered in the study, followed in descending order of influence by the exposure time, the shot size, degree of shot overlap, and finally the angle of inclination of the shot.

These findings contribute to the body of knowledge of LSP applications, as a virile surface treatment process for laser peening engineers and technologists to control the development and enhancement of residual stresses by guided adjustments to the LSP input parameters. With the development of verified and validated FEM-based numerical models as done in this study, more robust studies on LSP can be carried out as an alternative to the rigors and costs associated with experimental studies.

## Figures and Tables

**Table 1:** LSP simulation parameters.

Parameter	Value
Laser Power Intensity	6 GW/cm^2^
Peak Pressure	4 GPa (approx.)
Coverage	20 spots/mm^2^
Spot Size	0.6 mm
% Overlap	83.3%
Step Time	100 ns
Stress Damping Step Time	500 ns
Number of runs	1 × 2

**Table 2:** Physical properties of X12Cr steel material [30].

Property	Value
Density (kg/m^3^)	7700
Tensile Strength (MPa)	950
Yield Strength (MPa)	800
Elastic Modulus (GPa)	204.2
Poisson’s Ratio	0.30
Specific Heat (J/kg/k)	490
Thermal Conductivity (W/m^2^)	2.06
Shear Modulus (GPa)	77
Strain Hardening Modulus (MPa)	300
Damping Factor	0.05

**Table 3:** Chemical properties of X12Cr steel material [31].

Element	C	Si	Mn	Ni	P	S	Cr	Mo	V
Percentage Composition	0.08–0.15	Max 0.35	0.50–0.90	2.00–3.00	Max 0.025	Max 0.020	11.0–12.5	1.50–2.00	0.25–0.40

**Table 4:** Mie-Gruneisen equation of state parameters for X12Cr steel alloy.

*C_o_* (m/s)	*S*	Γ
4313.65	1.4545	2.06

**Table 5:** Summary of the comparison of the simulated and experimental results.

Parameter	Simulation	Experiment	Variation
Surface CRS (GPa)	−0.48	−0.46	3.6%
Maximum CRS Residual (GPa)	−0.715	−0.77	7.1%
Maximum depth of CRS (mm)	0.67	0.64	4.5%

**Table 6:** Hierarchy of influence of LSP input parameters.

	Input Parameter	% Probability Coefficient	Decreasing Hierarchy of Influence	
A	Shot Angle	0.5	Shock Intensity	0.96
B	Overlaps	0.71	Exposure Time	0.92
C	Shot Size	0.82	Shot Size	0.82
D	Shock Intensity	0.96	Overlaps	0.71
E	Exposure Time	0.92	Shot Angle	0.5

## Data Availability

Not applicable.
